# Cancer cell plasticity and therapeutic resistance: mechanisms, crosstalk, and translational perspectives

**DOI:** 10.1186/s41065-025-00564-8

**Published:** 2025-09-26

**Authors:** Saeid Ghorbian

**Affiliations:** Department of Bilogy, Ta.C., Islamic Azad University, Tabriz, Iran

**Keywords:** Cancer plasticity, Cancer stem cell, Tumor heterogeneity, Drug resistances

## Abstract

**Abstract:**

Resistance to targeted cancer therapies is a significant barrier to favorable treatment outcomes. Malignant cells can tolerate and resist drug treatments due to their biological flexibility. Specifically, slow-cycling drug-resistant cells may achieve permanent resistance to the treatment or restore sensitivity upon cessation of therapy. Enhancing cancer treatment methodologies necessitates a deeper understanding of the adaptability of tumor cells. Drug resistance and cellular heterogeneity are closely associated with cancer cell adaptability. Alterations in cellular signaling, interactions with the tumor microenvironment, and genetic and epigenetic alterations are all implicated. Analyzing these pathways will enhance our understanding of how cancer cells evolve and evade treatment. Two effective strategies to address cancer cell adaptability are to target specific biological pathways and to employ combination therapies. The progression of cancer therapy methodologies relies on comprehending and exploring the concept of cancer cell adaptability. Understanding tumor heterogeneity and drug resistance necessitates identifying the cellular, molecular, and genetic processes that govern cancer cell plasticity. This understanding enables the development of more personalized and effective cancer therapies, leading to improved treatment outcomes.

**Clinical trial number:**

Not applicable.

## Introduction

The emergence of new advancements in cancer treatment poses a considerable challenge to researchers and doctors in overcoming the different treatment resistances connected with this disease. Transformation mechanisms are necessary in addition to genetic changes that lead to resistance [1]. Enhancing the malleability of tumor cells leads to their conversion into a phenotype resistant to medicines. These robust cells constitute a layer characterized by a decreased growth rate. Post-treatment, these cells may either regain susceptibility to the medication or acquire enduring resistance. Understanding the processes of cellular plasticity provides innovative and enhanced strategies for cancer treatment [2]. Additional inquiry will yield more effective and pragmatic solutions to tackle these challenges. The adaptability of cancer cells allows them to alter in reaction to their environment, resulting in an increase in diverse tumors and resistance to treatment [3, 4]. The flexibility of cancer cells is associated with the process of epithelial-mesenchymal transition (EMT) and the characteristics of stem cells [5]. These pathways are integral to tumor formation and progression, complicating the nature of cancer and the therapeutic challenges it presents [6].

Despite advancements in targeted therapy, the capacity of cancer cells to adapt and develop drug resistance remains a substantial challenge in cancer treatment. Notwithstanding progress in effective targeted cancer medicines, treatment evasion continues to pose a significant challenge [7]. Historically, research has concentrated on genetic resistance, including mutations that disrupt drug binding or activate alternative signaling pathways [8]. These resistances arise from the selection of pre-existing uncommon genetic variants or the appearance of novel mutations following the initiation of treatment [9]. Recent evidence highlights the significance of non-genetic processes in treatment resistance. A minor fraction of cancer cells can endure despite the most effective therapies. These cells, called minimum residual disease (MRD), may lead to cancer recurrence and ultimately develop drug-resistant genetic alterations [10, 11].

**Fig. 1:**
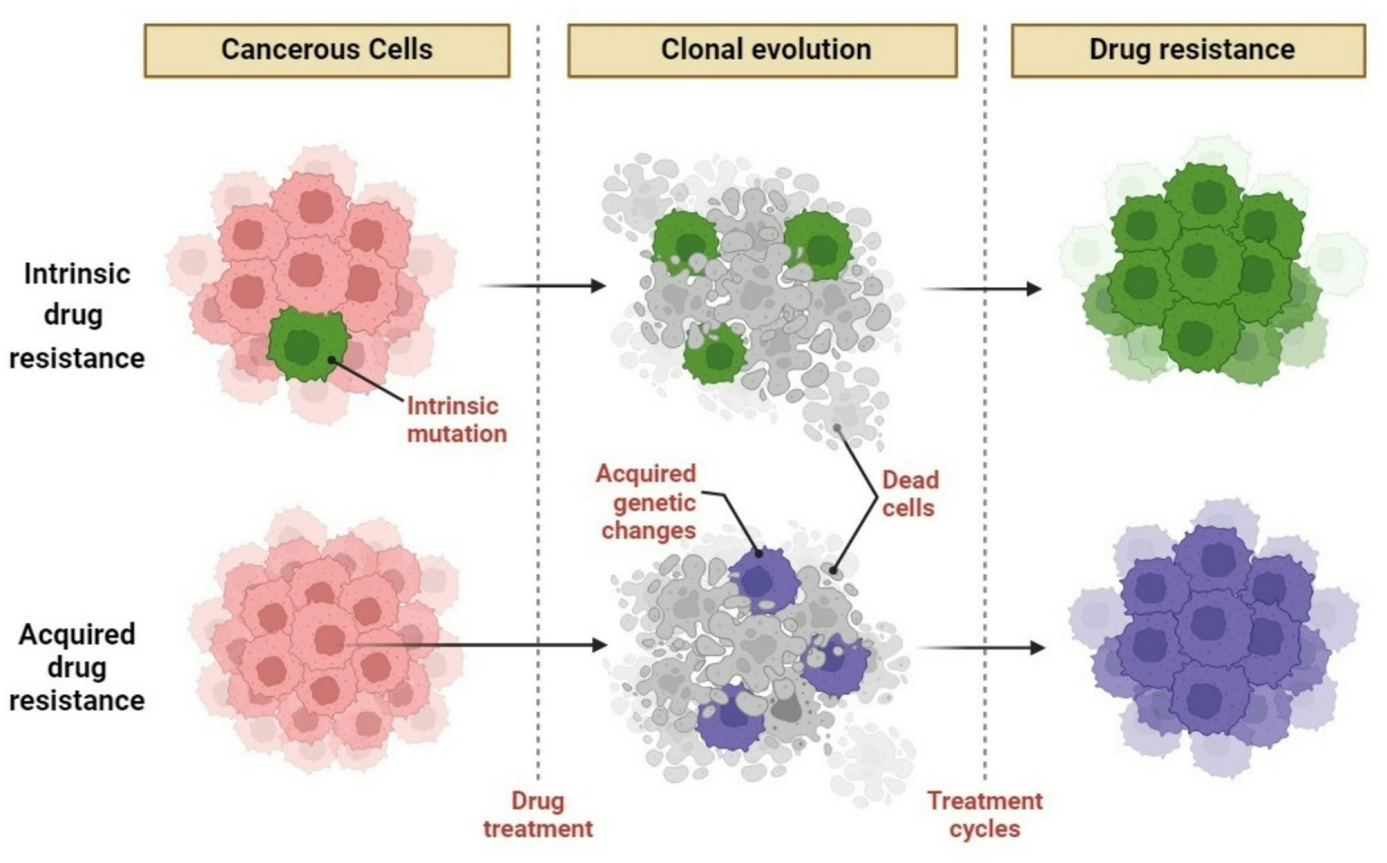
The pathways of drug Resistance. **1**) Pre-existing cell (green) that develop intrinsic drug resistance while undergoing treatment. **2**) Drug resistance developed. The cells can withstand the first treatment by gaining more genetic modifications (blue) that indicate resistance to treatment cycles

Cellular plasticity, or phenotypic flipping, allows cells to adjust to environmental alterations by assuming several morphologies (Fig. 1). In cancer, this capability enables tumor cells to reversibly transition into identities independent of drug-targeted pathways [12]. Focusing on this plasticity presents a promising approach to avert the emergence of drug-resistant cells and improve the efficacy of current treatments before developing new resistance mutations [13].

Investigations examine cellular plasticity as a mechanism to evade targeted therapy in several malignancies. The intricate epigenetic and transcriptional mechanisms that govern cellular plasticity and the crucial influence of the tumor microenvironment in promoting these phenotypic alterations highlight the urgent need for innovative therapeutic strategies to tackle this plasticity effectively. Comprehending cellular plasticity is essential for formulating a more efficacious therapeutic approach, as this characteristic is pivotal in cancer adaptability and treatment resistance [16]. Cancer cell plasticity is crucial for metastasis and tumor heterogeneity, enabling tumor cells to alter their phenotypes and evade terminal differentiation [17]. This characteristic disrupts successful cancer care by modifying tumor response and inducing treatment resistance. Cellular plasticity encompasses mechanisms such as EMT and associated signaling pathways, which underscore its significance in tumorigenesis and differentiation [18, 19]. The fundamental plasticity processes encompass the depletion of particular transcription factors and the development of stem and progenitor cell traits. Moreover, epigenetic reprogramming is crucial in cancer progression, exemplified by hypoxia-induced methylation alterations, highlighting a complex interaction among various factors affecting cancer development [20–22]. It also highlights the significance of tumor heterogeneity in fostering plasticity and the multiple mechanisms through which cellular alterations transpire. It underscores the importance of cellular plasticity in the development of drug resistance and explores its intricate functions, ranging from transient drug tolerance to permanent drug resistance. Data analysis will facilitate the identification of potential biomarkers and formulating innovative strategies to mitigate the effects of cellular plasticity on treatment efficacy [23]. Given the unresolved questions and prospective research avenues, this review advocates for a multifaceted approach to investigate and address cellular plasticity.

### Mechanisms of plasticity and resistance

#### Cellular mechanisms in cancer cell plasticity; EMT/MET

Grasping the significance of phenotypic plasticity is essential for understanding cancer’s development, progression, and therapy responses at the cellular level. Research continues to ascertain the determinants of the shift from hierarchical organization to phenotypic plasticity in cells. Nonetheless, there remains an urgent necessity for a more thorough comprehension of the unique fingerprints and fundamental mechanisms that orchestrate the dedifferentiation processes [24, 25]. The comprehensive understanding of phenotypic plasticity’s contribution to diversity within and among tumors continues to be an area of active investigation. The concept of phenotypic plasticity can expose unexpected vulnerabilities in the complex architecture of cancer, underscoring the significance of identifying and using aspects of plasticity to develop innovative anticancer therapies. The activation or inactivation of particular transcription factors dynamically modulates the differentiation processes in healthy cells and tissues [26]. The parallels among wound healing, development, and cancer indicate that specific variables can induce alterations in cellular activity, resulting in plasticity in both normal and malignant cells. EMT is crucial in cancer biology when cells transform from an epithelial to a mesenchymal phenotype. This process involves intricate epigenetic alterations, including cytoskeletal rearrangement, the creation of front-back polarity, and the breaking of intercellular connections. Conversely, MET signifies the reverse sequence of this evolution ( [27]– [28]). Key contributors to these transitions include microRNAs and essential signaling pathways, such as TGF-β, WNT, Notch, and Hippo, alongside transcription factors like Snail, Slug, Zeb1/Zeb2, and Twist ( [29]– [30]).

Transcription factors that belong to the Snail and Twist families activate genetic pathways that lead to substantial transcriptional alterations. Snail/SNAI1, Slug/SNAI2, and Smuc/SNAI3 suppress transcription and are integral to essential physiological processes, including mesoderm development and cell motility. Moreover, many transcription factors can initiate EMT, with their expression profiles varying according to tissue type or malignancy characteristics [31, 32]. MicroRNAs regulate these processes by suppressing or enhancing the expression of epithelial markers. Comprehending the intricate interactions among these components elucidates the sophisticated regulation of EMT, wherein diverse elements converge to influence cellular traits and behaviors [33].

Understanding the development of carcinoma has led to a growing body of evidence suggesting that the survival of cancer cells relies heavily on their ability to undergo cellular transitions, such as EMT. EMT is a fascinating process in which cancer cells originating from epithelial tissues undergo a remarkable transformation, enabling them to acquire a migratory and invasive behavior. This newfound ability allows cancer cells to escape from their original location and venture to distant sites in the body. It enables cancer cells to separate from their initial surroundings and migrate to different body areas, where they can potentially develop secondary tumors [34, 35].

Nevertheless, maintaining the mesenchymal phenotype is not always essential. It is fascinating how cancer cells can undergo a mesenchymal-to-epithelial transition (MET) when they reach a metastatic site. MET may enable cancer cells to thrive and develop in their new surroundings, potentially resulting in long-lasting metastases. Understanding the shifts in cell dynamics, notably the transition from mesenchymal to epithelial states, is crucial in comprehending how cancer cells can effectively adjust to various bodily environments [36, 37].

MET and EMT are controlled by various signaling pathways, including receptor tyrosine kinases (RTKs), TGF-β, Wnt, and Notch. It is important to note that TGF-β signaling, which is active in cancer cells, plays a crucial role in regulating the processes of EMT and MET. Transcription factors that support EMT, including Snail, ZEB, and Twist, can inhibit MET and induce a mesenchymal phenotype. Epigenetic modifications and microRNAs also significantly contribute to the regulation of these processes and aid in their management [38–41].

Developing novel and efficient therapies requires a more profound comprehension of these mechanisms and their interplay. Highly adaptive cancer cells are more prone to acquire medication resistance and evade treatment plans. MET targeting is investigated as a possible treatment approach. Preclinical and clinical studies have demonstrated encouraging outcomes for research aimed at MET-related molecular pathways, including TGF-β signaling and RTKs [42–44].

### Dedifferentiation

Cellular plasticity entails the conversion of cells from one lineage to another, referred to as neuroendocrine differentiation. This differentiation was observed in instances of prostate cancer and non-small-cell lung cancer (NSCLC). Comprehending the biology of prostate tumor proliferation is essential for formulating effective therapies. This instance emphasizes the targeting and reducing androgens critical to the androgen receptor (AR) signaling pathway. Studies demonstrate that genetic modifications and diverse therapeutic strategies can lead to neuroendocrine differentiation and treatment resistance [45, 46]. The genetic changes observed in adenocarcinomas persist intact following recurrence in neuroendocrine prostate cancer (NEPC). Moreover, stimulating neuroendocrine differentiation via androgen deprivation therapy can result in a dynamic transformation that enables the return of differentiation to an AR-driven state [47–50].

It is worth noting that tumor cells in prostate cancer can differentiate towards a neuroendocrine fate, as revealed by tracing analysis. However, cancer cells can develop resistance and continue proliferating. Cells in lung and prostate cancers resistant to androgen receptor signaling no longer depend on this signal. NEPC tumors display neuroendocrine characteristics such as decreased AR signaling, deletion of RB1 and TP53 genes, and expression of synaptophysin and chromogranin A. It is interesting to note that in both lung and prostate cancers, tumors lacking the TP53 and RB1 genes are more likely to differentiate into small-cell tumors. This suggests that silencing these genes might be a key genetic event promoting differentiation towards a neuroendocrine identity. The allelic inactivation of TP53 and RB1 was previously experienced by their common ancestor, according to a clonal study of lung adenocarcinomas that progressed to SCLC. This implies that the complete deactivation of both genes may be a reliable indicator for developing a neuroendocrine phenotype [51–53].

Nevertheless, prior research suggests that the loss of TP53 and RB1 alone cannot trigger neuroendocrine differentiation in adenocarcinomas or affect their responsiveness to EGFR-TKI inhibitors. Furthermore, it is worth noting that gene inactivation is observed in non-neuroendocrine tumors. This suggests that the range of mutations and the original type of cancer cells play a crucial role in determining the probability of tumor cells transforming into neuroendocrine cells [54, 55]. Understanding the plasticity of melanoma is critical in the field of cancer biology. Researchers have identified two main transcriptional programs that play a role in the differentiation stages of melanoma. The first program, known as the proliferative phenotype, is characterized by microphthalmia-associated transcription factor (MITF) and low expression of AXL. These markers are essential in determining the state of the cancer. The cancer cells exhibit a more differentiated and epithelial-like appearance in this phenotype. MITF activates differentiation-related genes, such as permelanosomal protein, dopachrome tautomeraseو, tyrosinase, and melan-A. Upstream activators frequently present at high levels in the proliferative phenotype, including SOX10, PAX3, EDNRB, and CREB, impact MITF regulation. The complexity of this regulatory network highlights melanoma’s flexibility and the role transcriptional programs play in its growth and differentiation. Comprehending these indicators and modulators offers a significant understanding of the molecular foundations of melanoma and opens up possible treatment paths [56–60].

### Cancer stem cells

**Fig. 2:**
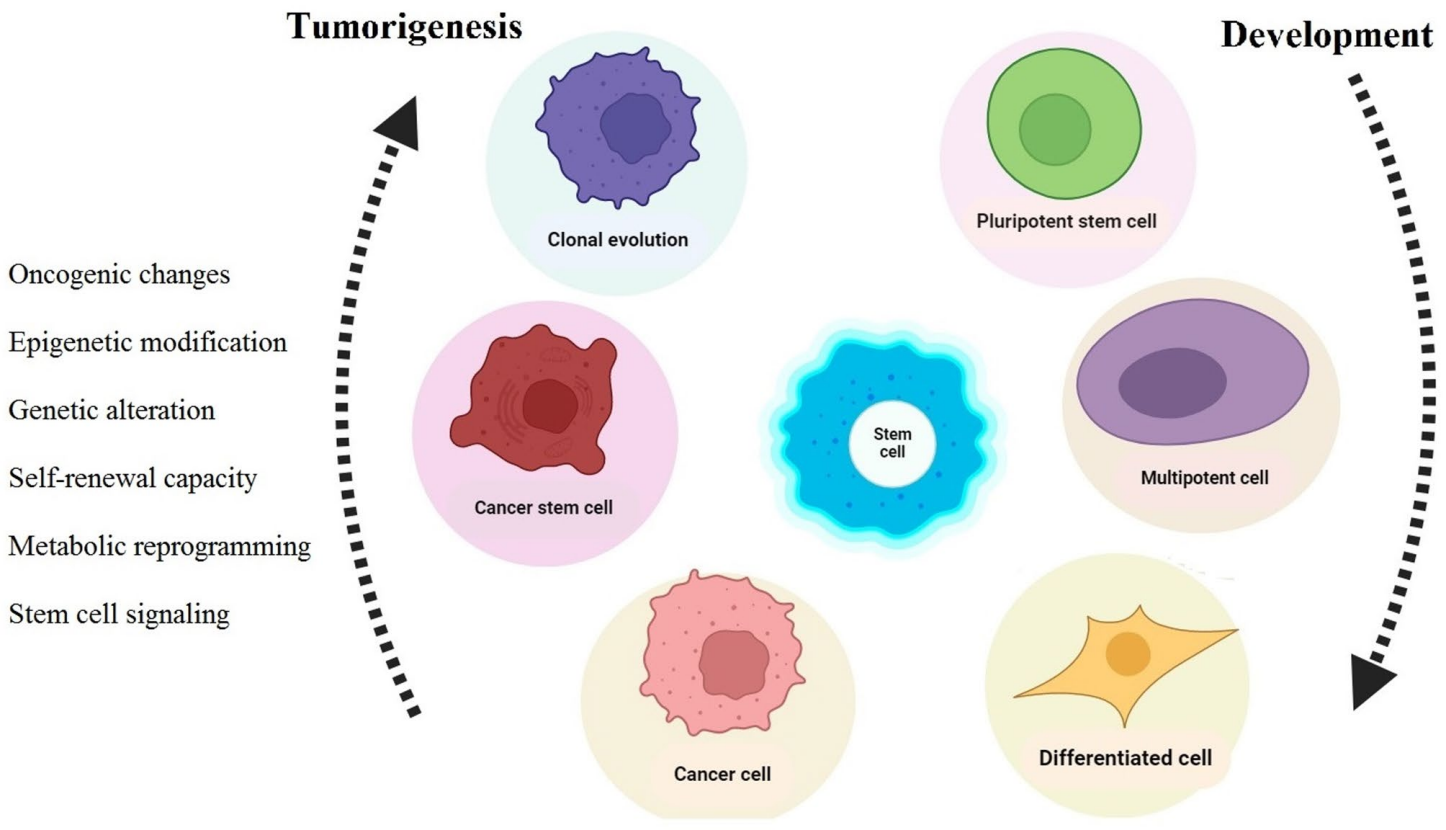
The development of CSCs and genetic rewiring. Diagrams show the dynamic changes that occur depending on the kind of cell throughout early development and carcinogenesis. All cell types can be developed from pluripotent stem cells, and these cells can eventually become fully differentiated adult tissues. Tumorigenesis, a process that disrupts the normal course of cell development, is facilitated by a complex interplay of epigenetic modifications, stem cell signaling, genetic alterations, oncogenic changes, and metabolic reprogramming. The process of tumorigenesis entails the differentiation of the cell state into CSCs, dysregulated stem cell-like entities. These are plastic cells that can self-renew capacity and specialize into numerous types of cancer cells

Cancer Stem Cells (CSCs) are a unique subpopulation of tumor cells with stem cell-like characteristics, such as the capacity to self-renew, produce different types of cells, and differentiate involved in tumor growth. These cells are crucial for the development and progression of tumors because they use their innate mechanisms of differentiation and self-renewal to promote malignancy. According to the model CSC concept, a particular group of cancer cells can self-renew, multiply, and change into different forms, resulting in varied appearance and antigen expression within the original tumor. This model illustrates a cellular hierarchy within each distinct tumor. For these cells to experience pathogenesis, it is necessary to interrupt the self-renewal process [61, 62]. Specific genes and communication pathways carefully control self-renewal in healthy cells. However, in CSCs, the regulatory mechanisms are compromised, leading to uncontrolled tumor cell growth while maintaining the ability to multiply. Understanding the disruption in the self-renewal pathway is crucial in combating tumors’ persistent resilience and growth. It is imperative to focus on targeting CSCs to develop effective therapeutic strategies. Within advanced tumors, “tumor stemness” revolves around the remarkable capacity of tumor cells to undergo self-renewal and generate the vast array of cellular elements that constitute the tumor mass [63, 64]. Understanding this process is made more complex by the EMT mechanism, which triggers mesenchymal traits and is linked to the activation of stem cell markers and an increased ability to form mammospheres, defining characteristics of mammary epithelial stem cells [65]. Activating EMT programs enables cancer cells to obtain CSC properties, enhancing metastasis and invasion. These cells gain the ability to detach from the primary tumor, infiltrate the bloodstream, and spread to distant sites. Moreover, cells exhibiting CSC characteristics rely not solely on particular programs like EMT. Alternatively, they may also emerge from random transitions. Research indicates that under specific conditions, certain cells inside the mammary epithelium can autonomously convert into stem cells and exhibit traits akin to cancer stem cells (CSCs). This transition enhances tumor growth capacity, and the adaptability of tumor cells presents opportunities for successful treatment techniques [66] (Fig. 2).

Non-CSCs in breast cancer cell cultures can rapidly convert into a CSC state, indicating that these transformations stem from the cells’ intrinsic flexibility [67]. Prior research has demonstrated that non-tumorigenic breast cells can withstand cellular change. Nonetheless, the rate of change from non-CSCs to CSCs in breast cancer remains ambiguous [68]. Multiple factors influence the formation and sustenance of cancer stem cells inside the tumor microenvironment. These factors allow the cells to demonstrate self-renewal, differentiation, survival, metastasis, and the possibility of cancer recurrence. It regulates cellular communication and signals that dictate cellular adaptation. In this environment, diverse biological constituents, including mesenchymal stem cells (MSCs), cancer-associated fibroblasts (CAFs), immune cells, and exosomes, are pivotal in influencing the plasticity of CSCs. Specific substances generated by immune cells, including macrophages, can convert non-cancer stem cells into cancer stem cells, enhancing their colony formation capacity. The plasticity exhibited in malignancies is regulated by many signaling pathways predominantly influenced by fibroblasts and CAFs. The pathways encompass Notch, IGF-II/IGF1R, c-Met/FRA1/HEY1, and FAK [69–72].

Recent investigations indicate that transitioning from neuroepithelial to mesenchymal cells in breast, ovarian, and pancreatic cancers is a slow and partial process rather than an abrupt change. Attributes in appearance and function evolve throughout time in different forms of malignancies. The gradual transition to a mesenchymal state is referred to as partial EMT. This discovery augments our understanding of cancer biology and underscores the need for more targeted therapy strategies that account for the dynamic changes in cancer cells [73, 74].

### Epigenetic modification in cancer cell plasticity

A critical factor in cancer cells’ flexibility is the widespread alterations in genome methylation patterns that show significant disruptions in epigenetics. The primary mechanism causing these alterations is the intricate interaction between external cues and the regulation of gene expression, namely by increasing the accessibility or compactness of chromatin. Furthermore, the epigenetic context may result in improper cellular reprogramming or differentiation arrest, altering the chromatin landscape’s openness or closeness. According to observations made in cancer genomic studies, cancer genomes may exhibit a bivalent chromatin state, defined by active and repressive markers close to specific genes.These characteristics, which enable the cells to constantly self-renew and undergo phenotypic change, may be preserved in CSCs, promoting the cells’ development and proliferation. A comprehensive comprehension of these epigenetic modifications is essential as it can enhance therapy options and provide a deeper insight into the intricacies of cancer [75]. Recent research suggests that epigenetic factors, rather than genetic ones, are more influential in the changes in cell identity induced by pharmaceutical interventions. These findings indicate that intricate genomic modifications in histone methylation patterns, resulting from the disruption of epigenetic regulators, are often associated with phenotypic anomalies. Furthermore, prostate cancer cells that have transformed into NEPCs may display modified cellular phenotypes and altered sensitivity to treatment due to the inhibition of EZH2 gene activity. These data indicate that EZH2 and other epigenetic factors may be pivotal in changing the identity of cancer cells and their response to treatment [76].

A notable characteristic of NEPC is the heightened activity of EZH2, the catalytic component of the Polycomb repressive complex 2 (PRC2). A significant feature of NEPC is the increased activity of EZH2, the catalytic element of the Polycomb repressive complex 2 (PRC2). This phenomenon has been evidenced across many animal models, cell lines, and clinical samples across multiple research platforms. In NEPC, EZH2 is essential for sustaining cancer cells’ aggressive and treatment-resistant nature [77]. Notably, EZH2 overexpression facilitated neuroendocrine development in a lung cancer mouse model, suggesting that EZH2 may be a universal mediator of this process. NEPC generally demonstrates dysregulation of the RE1 silencing transcription factor (REST), an additional epigenetic regulator. REST inhibits gene expression by recruiting co-repressors such as EZH2 [78]. The inhibitory function of REST in neuroendocrine development is noteworthy, as demonstrated by the increased expression of neuroendocrine markers, such as synapsin 1, following REST reduction in vitro. This REST activity is reflected in lung cancer, which converts neuroendocrine to non-neuroendocrine chemicals generated by Notch [79].

Various epigenetic alterations can impact the transcription of genetic sequences. These include chromatin remodeling, which is mediated by the SWI/SNF complex, and covalent histone modifications, including methylation and acetylation. These alterations are typically the result of genetic variations or other factors that impact the ever-changing chromatin landscape. Various factors are involved in these events, such as those responsible for methylation, demethylation, chromatin remodeling, insulation, and histone modifications. Biologists often observe a common occurrence in cancer where CpG islands in the promoter of tumor suppressor genes (TSG) become hypermethylated. This, along with a general hypomethylation landscape, can benefit oncogenes. The hypermethylated promoter was initially discovered in the promoter of the RB gene and subsequently observed in other types of cancer, such as renal cell carcinoma, where the VHL gene promoter is hypermethylated [80].

### Genetic mutations

Genetic abnormalities, such as mutations, chromosomal rearrangements, and other irregularities, primarily drive cancer occurrence. Advances in DNA sequencing technologies and extensive research have revealed that mutations in specific genes, specifically oncogenes and TSG, play a crucial role in developing numerous types of cancer. These genetic alterations not only initiate the transformation of normal cells but also enhance the diversity within the tumor and lay the groundwork for the development of various cancer subclones. Chromosome abnormalities, including aneuploidy and structural rearrangements like deletions, translocations, or amplifications, are frequently seen in cancer cells [81].

These large-scale genetic alterations can drastically modify cellular characteristics when paired with more subtle modifications like localized point mutations. While loss-of-function mutations can interfere with genes controlling the cell cycle and improve “stemness”-related features, both mutations can potentially change how cells behave in the context of cancer. Genes that control stem cell development or characteristics may also be impacted by flaws in DNA repair and replication processes, which may promote the flexibility of cancer cells. In hematologic malignancies, including acute lymphoid leukemia (ALL) and chronic myeloid leukemia (CML), leukemogenesis is induced by chromosomal translocations in hematopoietic stem cells. Genetic abnormalities in solid tumors, like colon cancer, can have significant impacts [82]. The absence of APC changes the Wnt signaling pathway, leading to increased cancer growth and invasion. These genetic modifications can also indirectly affect cancer plasticity. Furthermore, research has demonstrated that the loss of the Rb1 gene results in epigenetic disorders in retinoblastoma [83].

### Transcription factors (TF) in cell plasticity

Studying cell plasticity after drug treatment involves using transcriptional profiling, a crucial tool in this field. These analyses highlight the essential role of various reprogramming factors and lineage-specific transcription factors, such as the genes from the SOX family, in the ability to withstand and bypass therapy by switching lineages [84]. Research on basal skin cancer (BCC) examines the chromatin regions in cells that persist after drug treatment. These accessible regions have been found to contain a significant number of binding motifs for different transcription factors, including those from the SOX family. Based on the data, specific aspects and genes are crucial in determining the transition of cell identity in BCC following drug treatment. Prostate and lung cancer xenograft models and genetic models lacking Rb1 and p53 exhibit increased expression of SOX2, one of the transcription factors utilized to convert adult fibroblasts into induced pluripotent stem cells. SOX2 also stimulates neuroendocrine development. Accordingly, in vivo and in vitro models of prostate cancer that were treated with Sox2 short hairpin RNA suppression reversed the lineage transition and regained susceptibility to antiandrogen medicines [85–87]. It is worth noting that the study uncovered the involvement of MITF and SOX10 in both the development of melanocytes and the regulation of multiple cellular states related to drug resistance in melanoma patients. Intriguingly, SOX10 can facilitate the transformation of cell states in breast cancer by promoting a state resembling neural crest cells. In drug treatment, reprogramming and lineage-specific transcription factors influence cellular plasticity. Specifically, SOX2 is linked to the shift towards neuroendocrine differentiation, while SOX10 facilitates the transition to the neural crest state [88, 89].

#### EMT/MET-inducing transcription factors

The EMT process is regulated by transcription factors (EMT-TFs) that profoundly modify cell physiology. During EMT and its inverse, MET, cells experience significant alterations. They diminish intercellular adhesions, undergo a decline in epithelial polarity, restructure their cytoskeleton, and destroy basement membranes. The changes are influenced by numerous critical transcription factors, namely Snail1, Snail2 (Slug), Twist1, Twist2, ZEB1, and ZEB2. These molecules regulate gene expression by suppressing and binding to the promoter regions, especially those associated with cell-cell adhesion [90]. The Snail transcriptional repressor family interacts with the CDH1 promoter, suppressing E-cadherin production, an essential EMT component. The metastatic features of breast cancer are associated with the increase of Snail1 in the nucleus [91]. Moreover, tumor cells exhibiting metastatic HCC demonstrate markedly elevated levels of Snail1 compared to persons without metastatic HCC [92]. Furthermore, Snail2 is associated with other developmental processes, such as neural crest migration, gastrulation, and the initiation of epithelial-mesenchymal transition during cancer metastasis. The proliferation and progression of cancer are contingent upon Twist1 and Twist2, two constituents of the bHLH transcription factor family. Twist1 enhances gene expression by engaging with the SNAI2 promoter, facilitating EMT. This element is commonly seen in metastatic breast malignancies and is crucial in the initial stages of breast tumor development [93].

The ZEB family plays a crucial function in modulating cancer progression and participating in neural crest formation. ZEB1 and ZEB2 can attach to particular DNA regions referred to as bipartite E-box regions. This interaction results in the suppression of CDH1 promoter activity. Furthermore, they contribute to the upregulation of genes encoding matrix metalloproteinases (MMPs) and regulate the matrix remodeling process associated with EMT. Additionally, these pathways also encompass microRNAs. ZEB1 and ZEB2 establish a feedback loop that modulates EMT by binding to miR-200 promoters. The miR-200 family diminishes the levels of ZEB protein at the CDH1 promoter. The complex regulatory network underscores the mechanisms that regulate EMT and its significance in cancer progression [94, 95].

### Molecular mechanism of cancer cell plasticity

The molecular pathways that contribute to the plasticity of cancer cells are a critical focus of research in the complex field of oncology. This section analyzes the molecular dynamics of neoplastic cells. This trait enables cancer cells to circumvent treatment while enhancing their potential for metastasis and invasion. This comprehensive analysis establishes a basis for subsequent sections elucidating the ramifications of cellular plasticity and prepares for an understanding of the intricate characteristics of cancer cells. Comprehending the practical ramifications of molecular constituents demonstrates that cancer cell plasticity is not an isolated phenomenon but a vital component in the complex mechanisms of cancer progression and treatment resistance. The information in this section establishes a basis for future discussions on treatment methodologies and the diverse challenges faced in clinical environments. It is essential to fully understand the complexities related to this illness [96].

### Signaling pathways on cellular plasticity

#### TGF-β, smad, and non- smad signaling in EMT/MET

**Fig. 3:**
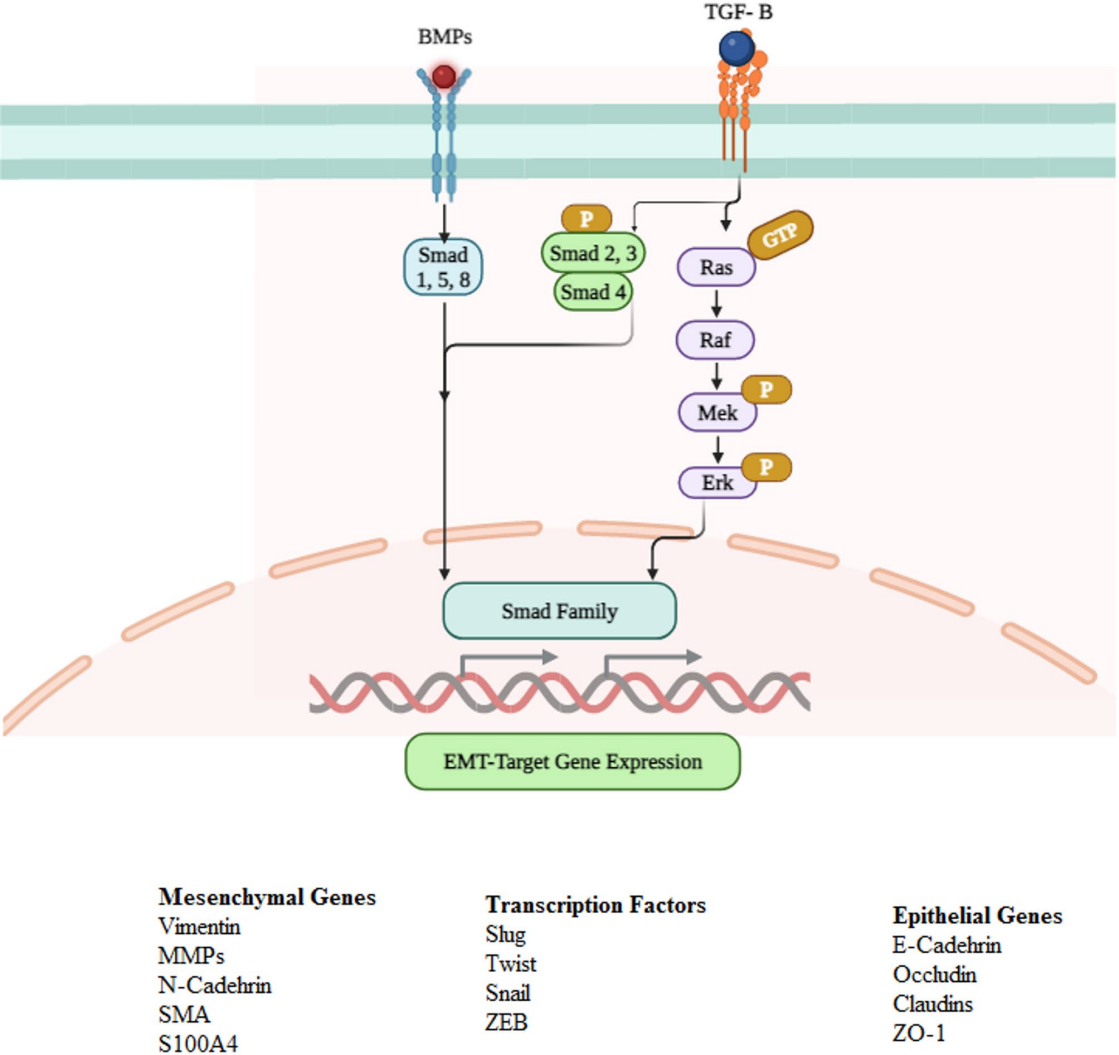
Illustrates the signaling pathways of EMT produced by growth factors in the development of cancer. TGF-β and bone morphogenic proteins (BMP) activate the expression of genes linked to EMT, such as transcription repressors and mesenchymal genes, by binding to the TGFRI/RII and BMPRI/RII receptors, respectively. Transcription activation can occur through the stimulation of the Ras/ERK pathway, which is dependent on Smads. Created with BioRender.com

TGF-β proteins function by using cell surface complexes, including two pairs of dual-specificity receptor kinases. They activate type I receptors, and ligands phosphorylate type II receptors when they bind. Smad proteins at their C-terminus, causing them to activate and separate from receptors. Though BMPs activate Smad1, Smad5, and Smad8, TGF-β activate Smad2 and Smad3. Still, TGF-β has been shown to activate Smad1 and Smad5. Once activated, these Smads join with Smad4 to create trimeric complexes that enter the nucleus and interact with transcription factors that attach to DNA to regulate target gene transcription. The DNA binding transcription factors and target genes that Smads regulate are diverse [97] (Fig. 3).

**Fig. 4:**
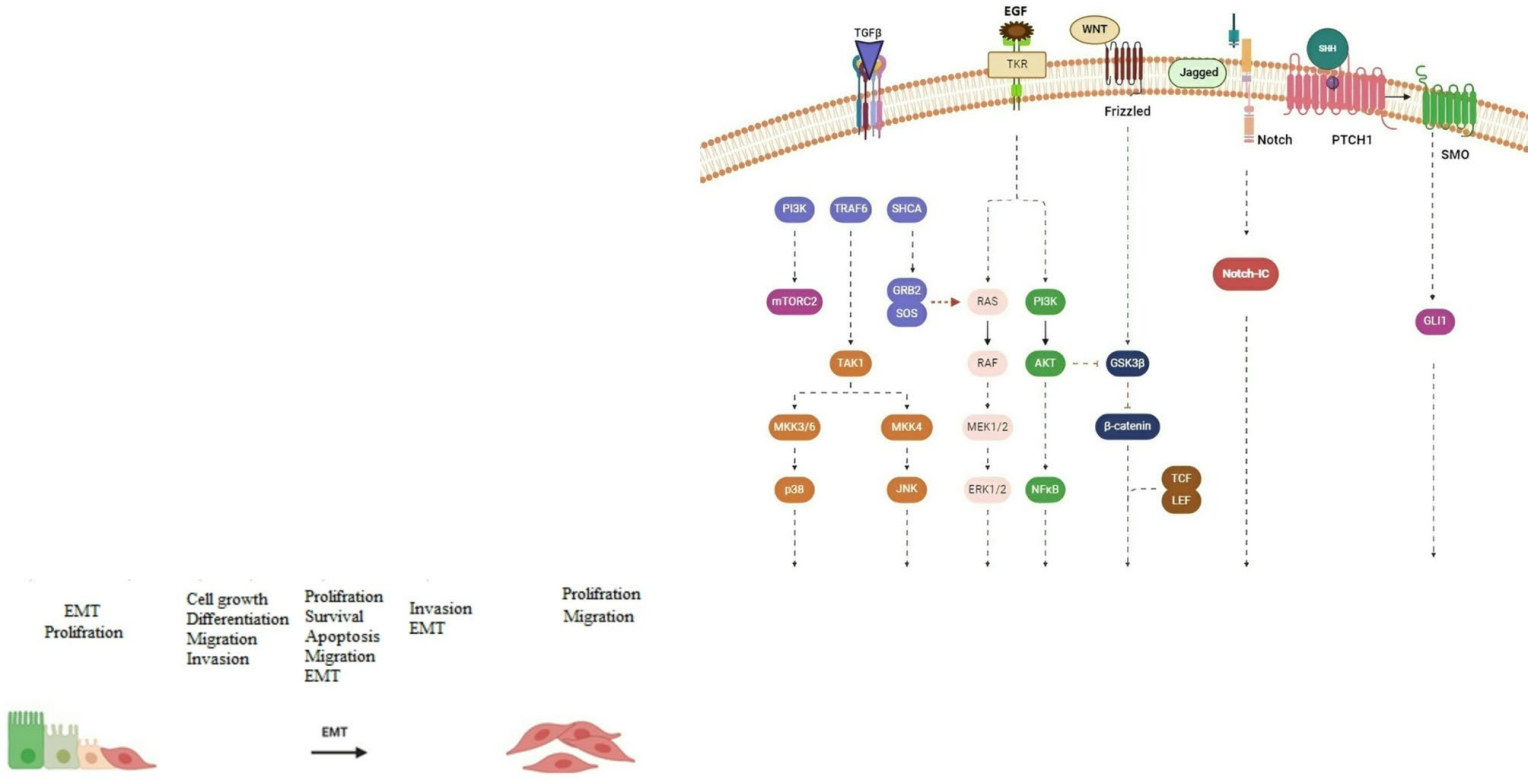
EMT programs signaling pathways. The regulation of EMT is governed by the common signaling pathways of TGFβ, WNT, PTCH1, NOTCH, and TRK. The signaling pathway culminates in the promotion of EMT by repressing genes that are markers of epithelial cells and activating genes that are typical of mesenchymal cells. The WNT signal pathway is triggered when a WNT-protein ligand binds to a Frizzled receptor, leading to the inhibition of glycogen synthase kinase-3β (GSK3β). This inhibition stabilizes β-catenin and prevents destruction. Following translocation to the nucleus, β-catenin interacts with the lymphoid enhancer-binding factor and T cell factor to initiate the production of genes associated with EMT. NOTCH signaling is activated when ligands from the Jagged family bind to the NOTCH receptor. This binding then starts a series of proteolytic cleavage events by the enzymes TACE and γ-secretase. As a result, the intracellular part of the NOTCH receptor is released to the cytoplasm. EGF can trigger EMT by activating RTKs, which in turn activate the RAS-RAF-MEK-ERK and PI3K signaling cascades that promote the development of the mesenchymal phenotype. TGF-β signaling is initiated when ligand binding, the TGFβ family of receptors. TGF-β can activate the PI3K-AKT, RAS-RAF-MEK-ERK, p38 MAPK, and JNK pathways. Simultaneous activation of these pathways can lead to the activation of the EMT program

Understanding Smad-mediated transcription relies heavily on the ability of the transcription factor to bind to DNA and the availability of the specific DNA sequence for Smad binding. Various activators and repressors, such as methyltransferases, histone acetyltransferases, deacetylases, and demethylases, are crucial in determining the repression and activation of Smad-mediated transcription. The collaborative character of Smad-mediated transcription serves as a foundation for signal disruption in gene regulation. The binding of Smad complexes with TCF, β-catenin, and LEF enables Smad to work along with Wnt signaling, whereas the interaction of Smad with CSL or NICD allows it to disrupt Notch signaling. Transforming growth factor (TGF-β) proteins can also stimulate non-Smad pathways, which result in non-transcriptional responses, while simultaneously influencing the regulation of genes regulated by Smad. TGF-β and BMPs activate Erk, JNK MAPK, p38, and signaling pathways. In particular, Erk MAPK signaling is triggered by the phosphorylation of Shc at Tyr by TβRI. Moreover, the TGF-β response involves the PI3K-Akt-mTOR pathway because BMPs and TGF-β activate Akt and PI3K. Ultimately, in the downstream pathway of the mTOR2 complex, TGF-β activates ROCK and RhoA. Uniquely, TGF-β and associated proteins can drive gene expression patterns and other cellular modifications required for EMT by combining these non-Smad pathways with Smad-dependent mechanisms for gene expression [98–100] (Fig. 4).

Among the many ligands in the enlarged TGF-β superfamily are three TGF isoforms (TGF-1, 2, and 3) and six BMP isoforms (BMP2 to BMP7). There are particular functions for each isoform in signaling activation. In some systems, especially cancer and fibrosis, TGF-1 is essential for regulating EMT. TGF-3 is active in palate development, although TGF-2 primarily controls EMT during cardiac evolution. BMP2 and BMP4, particularly BMP4, present in various tissues, have been linked to the induction of EMT in cancer. These isoforms’ significant prevalence in invasive epithelial tissues, as opposed to colon mucosa, fibrosis, and breast cancer, indicates their significance in revitalizing growth pathways. Curiously, BMP5 inhibits TGF-induced EMT, while BMP7 consistently inhibits EMT and primarily supports the epithelial cell phenotype. These discoveries highlight the impact of BMP isoforms on the development and symptoms of illness. It is worth mentioning that BMP signaling operates independently from TGF-β pathways, utilizing its own type II receptors instead of TGF-RII. Understanding the intricate connections between receptor types, signaling pathways, and ligand-receptor dynamics provides valuable insights into the intricate world of EMT induction and regulation. It highlights the significance of each element in the larger framework of growth and pathological phenomena [101, 102].

TGF-RII, a protein kinase that phosphorylates serine and threonine residues, activates SMAD2 and SMAD3 transcription factors. It facilitates the phosphorylation of TGF-RI, a crucial step in this intricate process. This process happens in the GS-rich domain of TGF-RI, resulting in the phosphorylation of Ser residues in the C-terminal domain of SMADs. This phosphorylation helps form a complex with SMAD4. When TGF-RI phosphorylates MH2 domains, it kickstarts the process of SMAD2/SMAD3 joining forces with SMAD4 and unraveling a nuclear localization signal. This unraveling is crucial for the R-SMAD/SMAD4 complex to enter the nucleus. Once inside the nucleus, SMAD complexes kickstart the transcription of genes associated with EMT. These complexes can interact with Snail1, suppressing the expression of genes that encode E-cadherin and occludin.

Additionally, they can bind to the SNAI1 promoter, promoting its transcription. The role of SMAD complexes in regulating EMT is not simple. It involves a complex interplay with the transcription factors ZEB and HGMA2, which are pivotal in controlling the expression of SNAI1, SNAI2, and Twist. This intricate dance of molecular interactions underscores the depth of our research in this field [103–105]. T.

GF receptors modulate SMAD-independent pathways and disrupt RTK signaling. The PI3K/Akt pathway is crucial in regulating EMT because TGF can activate PI3K either directly or indirectly. Studies have demonstrated that TGF-β triggers the activation of PI3K and Akt pathways in different types of cells. When PI3K is activated, it transforms PIP2 into PIP3, attracting and stimulating Akt. This phenomenon in cancer is linked to genetic alterations in the p110 subunit of PI3K and heightened synthesis of PIP3.

Furthermore, integrin activation can induce the phosphorylation of Akt by ILK. Akt2 activation leads to the phosphorylation of hnRNPE1, resulting in an upregulation of the translation of proteins associated with EMT. Akt enhances EMT in squamous cell carcinoma cells by activating NF-κB and upregulating SNAI1 gene expression [106–108] (Fig. 4).

#### Wnt signaling

The LRP and Frizzled receptors in the plasma membrane initiate the Wnt signaling process. A group of proteins, including Axin, GSK-3β, and APC, is responsible for phosphorylating beta-catenin and retaining it in the cytoplasm for proteasomal destruction when no signal is present. A change in signaling caused by Wnt ligands binding to Frizzled causes LRP6 to become phosphorylated and attract Axin and Disheveled to the plasma membrane. This mechanism blocks β-catenin phosphorylation and enables β-catenin to enter the nucleus, where it binds to TCF/LEF transcription factors to facilitate EMT. In addition to promoting EMT, the Wnt pathway causes Snail1 and Snail2 to stabilize and become active. This process is crucial in the development of invasive breast cancer and is linked to a reduction in E-cadherin and an increase in fibronectin. Furthermore, the WNT-β-catenin pathway induces treatment resistance in CRPC and basal cell carcinoma (BCC). Suppression of WNT signaling with substances like LGK-974 and LRP6 antibodies might decrease the number of remaining abnormalities and postpone tumor recurrence. The IL-6-STAT3 pathway inhibits the androgen receptor in PC, leading to EMT characteristics and neuroendocrine differentiation. Furthermore, activating the retinoid X receptor in melanoma plays a role in developing neural crest-like stem cells (NCSCs). By using the specific antagonist HX531, it is possible to target RXR and decrease the number of NCSC-like cells. This, in turn, enhances the responsiveness of melanomas to treatment [109–112] (Fig. 4).

#### Notch signaling

It significantly impacts the cell plasticity induced by drugs in cancer. Within this pathway, enzymes like γ-secretase and TACE play a role in degrading the Notch receptor, releasing NICD. NICD moves into the nucleus and interacts with CSL transcriptional repressor complexes, activating genes crucial in tumor proliferation. This pathway indirectly influences EMT by modulating various signaling cascades and controlling the expression of miRNAs. Furthermore, an excessive Notch expression within the vascular network can activate EndMT and deplete vascular endothelial cadherin. Furthermore, these investigations have demonstrated that restricting the activity of Notch1 can decrease the invasive characteristics and reverse the process of EMT in lung cancer. In summary, the intricacy of Notch signaling and its diverse impacts on controlling EMT are well established [113, 114] (Fig. 4).

#### The role of microenvironment in cancer cell plasticity

Tumors consist of more than just cancer cells; they also have an intricate connection with their surrounding environment, referred to as the tumor microenvironment (TME). This interaction has been the subject of significant interest in research and clinical studies. Within the TME, various cell types, such as tumor cells, immune cells, and stromal cells, interact complexly. The TME also consists of blood vessels and extracellular matrix (ECM). Study findings show that cancer cells can use intricate communication networks to regulate the behavior of both cellular and non-cellular components. These interactions between cells and their surroundings result in new phenotypes in non-malignant cells, promoting tumor growth. Moreover, these relationships may play a role in the development of tumors, their inherent variety, and their resistance to medication treatments [115]. Tumor cell plasticity enables tumor cells to alter their characteristics and adapt to ensure their survival, and the TME plays a crucial role in facilitating this interaction. Studies have demonstrated that the quantity of genetic mutations in cells within a tumor is significantly more than what is observed in laboratory cultures, indicating that the TME may contribute to genetic instability during the advancement of the tumor.

Furthermore, tumor heterogeneity might cause the reorganization of the TME, leading to the development of treatment resistance in cancer [116]. Recent studies have demonstrated that hypoxia can alter cell phenotypic and hence lead to medication resistance. Research findings indicate that hypoxic stress modifies EMT phenotypes in lung cancer and modifies EMT transcription factors, including SNAI1, SNAI2, and ZEB2. These modifications eventually result in resistance to cytotoxicity. Tumor hypoxia can be a risk factor for increased development of CSCs and metastasis, in addition to being one of the primary causes of medication resistance. Furthermore, cytokines that contribute to the tumor environment, including TNF and interferon-gamma, seem to make stromal cells more malleable [117].

The interaction between cancer cells and other stromal components, such as immune cells, endothelial cells, and fibroblasts inside the extracellular matrix (ECM), is essential in solid tumors. A network of blood vessels supports this interaction. This complex environment coordinates crucial processes in the progression of tumors and their response to treatments. The release of factors and interactions with the ECM regulates cellular adaptability to medicinal treatments. Cancer-associated fibroblasts (CAFs) play a crucial role in promoting treatment resistance in melanoma by directly interacting with cancer cells and releasing soluble mediators such as HGF [118].

Fibroblasts in areas with abundant supporting tissue demonstrate heightened MAPK activity, modifying the structure and stiffness of the tumor matrix. Consequently, melanoma cells might rapidly acquire resistance to the therapy. Mature fibroblasts secrete the protein sFRP2, which inhibits WNT signaling. This protein diminishes MITF synthesis and augments resistance to BRAF inhibition [119].

### The role of non-coding RNAs in cancer cell plasticity

Non-coding RNA (ncRNA) consists of molecules recognized for their inability or limited capacity to encode proteins. It is fascinating to learn that most human genes produce RNAs that serve functions beyond protein translation. Recent advancements in cutting-edge technologies and combining different scientific disciplines have unveiled an intricate signaling network formed by non-coding RNAs in human cells. These non-coding RNAs significantly impact various biological processes and are crucial for accurately controlling gene expression. Their various functions highlight the intricate levels of regulatory control that contribute to the vast network of genomic interactions. This finding not only broadens our comprehension of genetic regulation but also presents fresh avenues for investigating various biological processes at the molecular scale [120].

#### MiRNAs in cancer cell plasticity

MiRNAs, which are between 19 and 23 nucleotides long and non-destructive, are essential for controlling the expression of genes following transcription. These molecules attach to complementary sequences in target mRNAs to either cause or prevent mRNA instability. miRNAs are implicated in apoptosis, cell division, and proliferation. Research indicates that the disruption of miRNA regulation can act as either an oncogene or a tumor suppressor and is associated with many malignancies. Research indicates that cancer cells frequently undergo the EMT pathway by activating TGF-β [121]. TGF-β regulates EMT in advanced malignancies by increasing the activity of Snail, ZeB, and Twist transcription factors. Various microRNAs, including miR-200, miR-21, and miR-31, serve as possible mediators in the EMT pathway induced by TGF-1. Stimulating TGF-β enhances the production of miR-31 and miR-21, which specifically target TIAM1 and promote EMT. According to studies, TGF-β stimulates the EMT process, which is frequently present in cancer cells. By promoting the transcription factors Twist, Snail, and ZeB, TGF-β coordinates EMT in advanced malignancies. Many miRNAs, including miR-21, miR-200, and miR-31, may function as middlemen in the TGF-1-to-EMT pathway. TGF-β stimulation enhances the production of miR-31 and miR-21 to target TIAM1 and facilitate EMT. The miR-200 family, comprising miR-200B, miR-200 C, miR-200 A, miR-141, and miR-429, significantly regulates EMT, thereby successfully inhibiting tumor metastasis. These miRNAs diminish in invasive breast cancer cells and cells undergoing TGF-β-induced epithelial-mesenchymal transition. SIP1 and ZB1 mRNAs are transcripts of E-Cadherin, and miR-200 suppresses them to maintain E-Cadherin levels and epithelial shape. Modifying miR-200 in mesenchymal cells, SIP1 and Zeb1 establish a feedback loop that affects EMT and offers potential treatment avenues [122–126].

#### Long non-coding RNAs in cancer cell plasticity

Long non-coding RNAs (LncRNAs) have a considerable fraction of ncRNA sets, ranging from 200 pairs to 100KB. These chemicals, some of which are not yet functioning, may have a repressive or carcinogenic impact on different types of cancer.

#### LncRNA-HOTAIR & ANRIL

HOTAIR and ANRIL are prominent lncRNAs that substantially influence drug resistance, metastasis, and cancer. Early-stage breast tumors and metastases exhibit increased HOTAIR expression, recognized as a dependable prognostic indicator of malignancy. Inhibiting its expression may hinder the proliferation of cancer cells [127]. Moreover, ANRIL facilitates cancer progression by suppressing the expression of the P15 INK4B gene. These lncRNAs facilitate cancer progression by obstructing the function of genes that limit tumor growth. The complex interaction between lncRNAs and miRNAs is essential in governing cancer traits. Studies have shown that elevated temperatures contribute to EMT and the preservation of CSC populations in breast cancer.

**Fig. 5:**
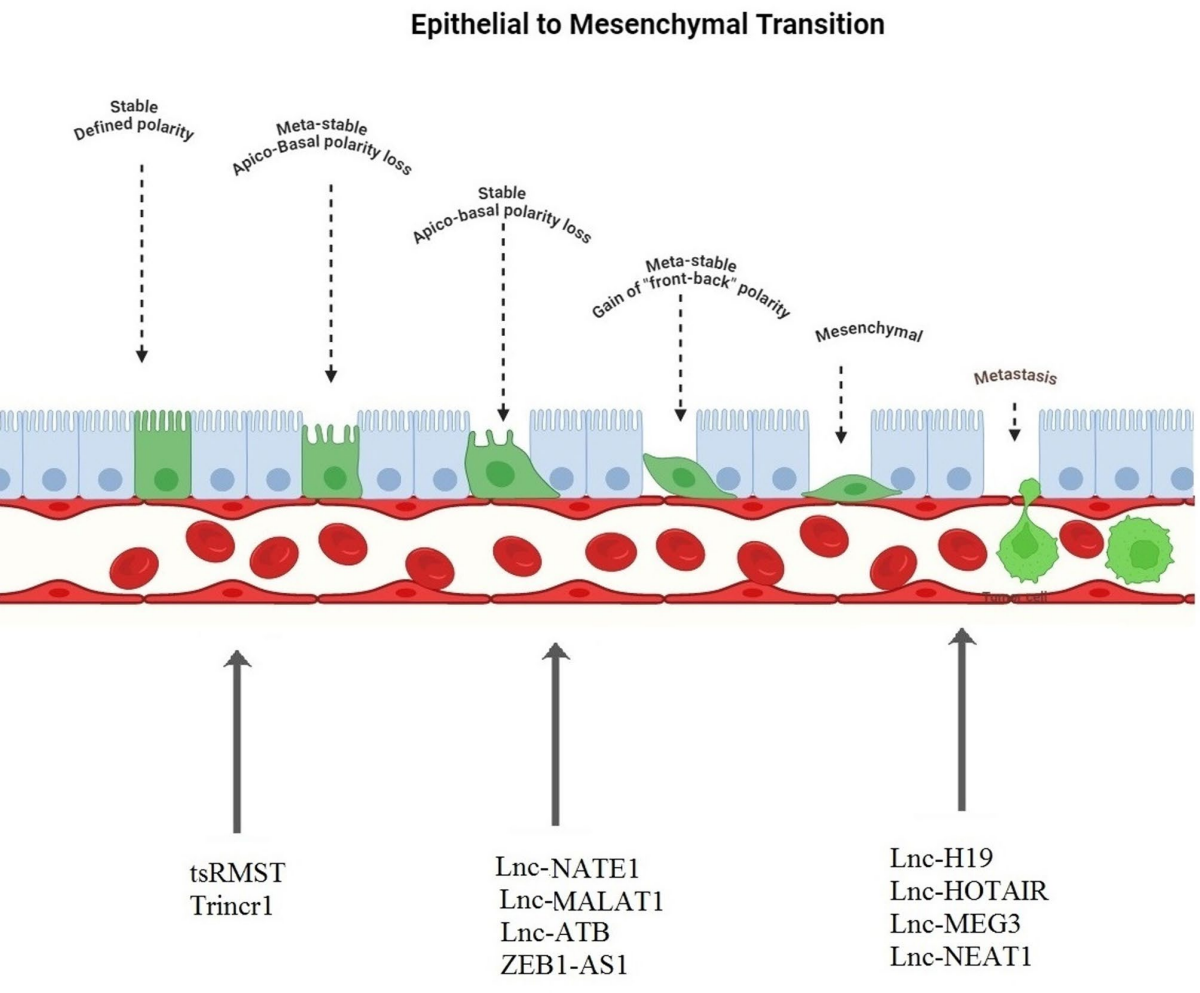
LncRNAs affect different EMT subtypes. Polarized epithelium cells functionally change into secretory, migratory mesenchymal cells during EMTs. Cell transition is characterized by a gradual decrease in epithelial markers and an increase in mesenchymal markers. The presence of mesenchymal and epithelial cell markers, as well as their lncRNAs, is demonstrated

In contrast, miR-34 A reduces the expression of HOTAIR in prostate cancer. This complex network regulates non-coding RNAs, genes linked to EMT, and the associated signaling cascades. It is essential in ascertaining the compatibility and phenotype of CSCs. Notably, HOTAIR, an important lncRNA in breast cancer, participates in the epithelial-mesenchymal transition process and sustains the population of cancer stem cells. This lncRNA is essential in controlling cancer-related mechanisms by influencing the expression of the HOXD10 gene and interacting with specific microRNAs, including miR-34 A. The links indicated that miRNAs and lncRNAs may be viable therapeutic targets (Fig. 5). Nonetheless, complexities and ambiguities regarding their function and efficacy persist, warranting more investigation [128–131].

#### LncRNA-MALAT1

MALAT1 is a lncRNA crucial in several stages of cancer initiation and progression. This RNA regulates numerous essential signaling pathways and mediates in various intracellular processes. Regulation of alternative splicing: Through engagement with proteins associated with alternative splicing. This chemical can also influence splicing patterns. This synthesizes several mRNA variants, which may alter proteins and cellular organelles. The MALAT1 gene interacts with transcription factors and proteins, either promoting or suppressing the transcription of essential genes involved in cancer progression. MALAT1 can alter chromatin structure by interacting with different chromatin complexes. This may lead to changes in gene availability and the regulation of their function. MALAT1 can affect cancer cell plasticity through its molecular actions by facilitating EMT, a crucial phenotypic change associated with increased cell proliferation and migration. Moreover, it can enhance cell proliferation, a hallmark of cancer cell spread, and facilitate the emergence of drug resistance, a significant challenge in cancer therapy [120, 132].

#### LncRNA-H19

H19 is a lncRNA that is expressed as an imprint. This lncRNA is improperly regulated in various malignancies and is involved in cellular mechanisms associated with cancer cell plasticity. H19 is especially engaged in the control of signaling pathways related to EMT, metastasis, and CSC. H19 increases EMT by altering the TGF-β signaling pathway, a key process regulator. H19 regulates genes in the TGF-β pathway, leading to EMT-related phenotypic alterations and cancer cell migration and invasion. Furthermore, H19 modifies the genes of signaling pathways linked to metastasis, improving tumor cells’ capacity to spread to different organs. H19 can control the production of invasive factors like MMPs, which aid in the breakdown of the extracellular matrix and promote the migration of tumor cells. Additionally, it promotes the survival of these cells by controlling the signaling pathways associated with the fundamental characteristics of cancer cells. Generally speaking, CSCs are recognized for their self-renewal capacity and strong resistance to anticancer therapies. Its impact on pathways like Wnt/β-catenin is essential for the differentiation and self-renewal of CSC and helps it survive [133–135].

#### LncRNA-UCA1

In certain cancer types, this specific molecule demonstrates abnormal expression. This lncRNA significantly enhances cancer cell plasticity by regulating EMT, treatment resistance, and metastasis. UCA1 engages with proteins and miRNAs within various pathways to promote these effects. MiRNAs, generally negative regulators of EMT, influence UCA1. UCA1 can enlist the endogenous inhibitors of epithelial-mesenchymal transition, miR-145 and miR-204. UCA1 enhances the expression of EMT-stimulating genes by reducing miRNA levels. This lncRNA modifies the phenotypic of cancer cells by upregulating mesenchymal genes and downregulating epithelial genes, thereby enhancing their motility and invasion.

Furthermore, it is crucial in facilitating the dissemination and invasion of cancer cells via its interaction with proteins associated with the Wnt and β-catenin signaling pathways. It facilitates the degradation of the extracellular matrix and the motility of tumor cells via regulating genes such as MMPs. UCA1 is crucial to cancer cells’ resistance to chemotherapy agents. It accomplishes this by using miR-16 and enhancing the synthesis of proteins such as P-glycoprotein [136–138].

#### LncRNA-TUG1

TUG1 regulates the expression of genes associated with EMT, metastasis, and therapeutic resistance, affecting the adaptability of cancer cells [139]. The interactions mediate the effects between TUG1, transcription factors like ZEB1 and Snail, and chromatin-modifying complexes like PRC2. TUG1 interacts with PRC2 and alters chromatin architecture, inhibiting epithelial gene expression and promoting mesenchymal gene activation. Moreover, the presence of ZEB1 and Snail amplifies the expression of mesenchymal genes while inhibiting epithelial genes, hence facilitating the migration and invasion of tumor cells [140]. TUG1 can augment the chemoresistance of cancer cells by upregulating the expression of multidrug resistance (MDR) genes. Moreover, TUG1 can engage with specific miRNAs that are involved in the regulation of drug resistance. These interactions reduce the efficacy of miRNAs and augment the expression of genes linked to treatment resistance [141].

#### LncRNA-MEG3

MEG3 acts as a tumor suppressor and is downregulated in various cancer types. MEG3 can reduce the expression of miR-155 and miR-21, microRNAs that inherently promote EMT [142]. These interactions lead to a decrease in the expression of genes linked to mesenchymal traits and an increase in the expression of genes linked to epithelial traits, suppressing EMT. Moreover, MEG3 augments the activity of transcription factors critical for preventing EMT, such as p53, and engages with these factors to reduce EMT. Furthermore, MEG3 can reduce the expression of genes associated with metastasis, including MMPs. These enzymes facilitate the degradation of the extracellular matrix and enhance tumor cell motility by inhibiting miR-21 [143].

#### LncRNA-NEAT1

NEAT1 (nuclear paraspeckle assembly transcript 1) is a lengthy, non-coding RNA that profoundly influences the adaptability of cancer cells. NEAT1’s functional mechanism involves multiple facets related to gene activity control, cellular structure and function modifications, and interactions with other elements that facilitate cancer growth. The signaling mechanisms of NEAT1 remain incompletely elucidated; nonetheless, significant pathways like PI3K/AKT, Wnt/β-catenin, and MAPK are likely to influence it. These pathways are essential for regulating cell division, growth, and migration. Through its interaction with components of these pathways, NEAT1 not only affects cancer progression but also modulates essential processes associated with cellular plasticity, thereby underscoring its importance in cancer biology [144, 145].

### Challenges in overcoming resistance

#### Tumor heterogeneity (Intratumor and intertumor) and cancer cells plasticity

This section examines cancer cell plasticity and tumor heterogeneity, illustrating how various cellular settings inside tumors facilitate cancer cell adaptability and plasticity. These insights establish a fundamental foundation for a deeper understanding and the progression of subsequent sections. Specific cancer types have been identified to harbor CSCs, signifying their capacity to differentiate into non-tumorigenic cells. The application of this paradigm to different cancer types and its treatment implications remains unknown. Deep sequencing is a sophisticated technique that offers a more thorough insight into therapy resistance and illness progression. Recurrence and metastasis account for almost 80% of cancer fatalities, underscoring the substantial ability of cancer to reappear and disseminate. Managing advanced stages of cancer is a considerable difficulty owing to the extraordinary capacity of cancer cells to adapt. This adaptation depends on epigenetic reprogramming and is affected by the tumor microenvironment. Tumor heterogeneity can be categorized as intratumoral and intertumoral heterogeneity [146, 147]. Intratumor heterogeneity refers to the diversity of tumor cells within a single tumor. Conversely, intertumor heterogeneity illustrates the genetic variations among people possessing the same tumor type. Two principal hypotheses have been proposed to elucidate this variability: the “clonal evolution” model, which posits that random genetic alterations confer a survival advantage to specific cells, and the “cancer stem cell-like” model, which underscores the significance of self-renewing cells in initiating and perpetuating tumors. The “CSC plasticity” concept posits that cancer stem cells can shift between undifferentiated and differentiated states in reaction to genetic and environmental stimuli [148–150]. EMT is a characteristic associated with an increased risk of tumor starts. The cancer microenvironment, comprising cytokines, growth factors, tumor-associated fibroblasts (TAFs), and related macrophages, affects the interactions between CSCs and the EMT, which entails cellular modifications resulting in the conversion to stem cells. The regulation is affected by tumor-associated macrophages (TAMs) and hypoxic conditions. This connection exacerbates medication resistance, facilitates the metastasis of cancer to other regions of the body, and contributes to disease recurrence [151, 152]. Research indicates that non-stem cancer cells can acquire stem-like characteristics mediated by factors such as ZEB1. Cell surface indicators are predominantly utilized to identify CSCs, demonstrating remarkable variety and adaptability among cancer types. Research indicates that many markers and genes, including the Wnt target gene and LGR5, are essential for this plasticity in colorectal cancer. A recent study has identified particular markers for metastatic cancer stem cells, further complicating the situation. Studies demonstrate that the concurrent activation of mesenchymal and epithelial genes amplifies the stem cell-like characteristics of cancer cells, observable through the formation of structures termed “tumorspheres.” Research suggests that cells in a partially EMT state may have enhanced tumor initiation capabilities akin to fully mesenchymal cells. This discovery challenges the relationship among the stemness of cancer cells, the partial epithelial-mesenchymal transition state, and the cancer stem cell and cancer evolution models. It underscores the necessity for a more thorough and integrated understanding of cancer heterogeneity [153, 154].

#### Drug resistance in cancer cell plasticity

We still have much to learn about these mechanisms. However, it is believed that these mechanisms follow a gradual process, where tumor cells initially shift from a state of drug tolerance to eventually developing a drug-resistant identity. Most studies investigating the survival procedures of slow-cycling cells have mainly been conducted in laboratory settings using the idea of drug-resistant persistence [155].

Research indicates that non-stem cancer cells can develop stem-like characteristics influenced by factors like ZEB1. Cell surface markers are primarily used to identify CSCs exhibiting significant heterogeneity and adaptability across various cancer types. Studies have revealed that specific genes and markers, such as the Wnt target gene and LGR5, significantly impact colorectal cancer (CRC) tumor plasticity. Following focused treatment of cancer cells, a range of outcomes may arise, such as partial tumor removal or cancer cell persistence. These changes typically arise from genetic mutations or cellular reprogramming [156, 157]. Under certain circumstances, the complete deactivation of TP53 and RB1 genes can make cells more susceptible to neuroendocrine differentiation. After treatment, the remaining cells typically experience a more relaxed life cycle, and the pathway targeted by the drug is suppressed. In addition, certain factors, like the histone-modifying enzymes and SOX family genes, have been found to play a role in reprogramming cells to develop drug resistance [158].

Understanding the intricate dynamics of the tumor environment involves studying the impact of cytokines secreted by cancer-associated fibroblasts (CAFs) and macrophages. CAFs release hepatocyte growth factor (HGF) and TGFβ, while macrophages produce tumor necrosis factor (TNF) and IL-6. Additionally, the level of oxygen plays a role in regulating cell plasticity. If treatment is stopped, cells resistant to the drug may activate a pathway that promotes cancer, return to their original characteristics, multiply, and regain sensitivity to the drug. The cellular phenotypic transition from drug sensitivity to drug resistance has been discovered in prokaryotic cells for the first time. Cells that are resistant to drugs can live even after being treated with antibiotics. Furthermore, these cells can resume their ability to multiply if the drugs are stopped, and they can re-establish a population that is susceptible to the drugs. This suggests that phenotypic alteration does not transpire via genetic modifications. Drug-resistant stabilizers (DTPs) are subpopulations of cancer cells that have been found to withstand exposure to fatal medications after therapy briefly. Curiously, this state of being resistant to drugs is temporary yet remains genetically consistent. Moreover, these cells originate sporadically from a cell population that is constantly changing variably. In addition, comparable drug-resistant quiescent cells have been discovered in living organisms [159].

These trials illustrate that establishing a state of slow cycling can effectively foster medication tolerance. This research reveals that all drug-tolerant cell morphologies, even in a condition of slow cycling, include unique resistance mechanisms that transcend genetic modifications. Prolonged culture of DTPs can lead to the emergence of several resistance mechanisms, including irreversible genetic changes frequently seen in patient samples that result in the reactivation of the drug-targeted pathway. Consequently, this may entail several stages, including the following: Following prolonged pharmacological intervention, cancer cells reversibly reconfigure their transcriptome to adopt a slow-cycling phenotype. Subsequently, they recover the ability to proliferate and ultimately resist other epigenetic alterations, diverting them from the target medication [160].

Genetic alterations may trigger this process. This indicates the existence of a drug-resistant, slow-cycling state that is independent of tumor type or treatment protocol, presenting a potential target for therapeutic intervention. It is essential to recognize that most of these studies were performed in laboratory environments, and subsequent research should occur in home contexts. The formation of a novel cellular identity during pharmacological intervention can be ascribed to the environmental conditions of the tumor cell of origin and the epigenetic and transcriptional alterations associated with this process [161].

#### Exploring the phenomenon of tumor cell plasticity

Comprehending the influence of tumor cell plasticity on drug resistance is essential for enhancing treatment outcomes. Formulating pharmaceuticals that precisely target these processes is promising in this context. Multiple techniques have been proposed, including impeding plasticity development by inhibiting the fundamental molecular pathways, eradicating drug-resistant tumor cells to focus on cells with an alternative identity, and reversing the process to reinstate the original condition. Employing alternating doses to inhibit essential pathways after exposure to carcinogenic agents, in conjunction with targeted therapy, immunotherapy, radiotherapy, or chemotherapy, while focusing on slow-cycling cells and disrupting signaling pathways, can facilitate the creation of a novel cellular identity. Intermittent treatments may impede the emergence of enduring resistance in cells. Formulating intermittent dose regimens in clinical practice is challenging due to the simultaneous emergence of many resistance mechanisms [75].

### Harnessing cellular plasticity

#### Integrating combined treatments

Utilizing combined and alternating treatment regimens as an innovative strategy to avert medication resistance offers advantages over monotherapy methods. These strategies can effectively reduce the development of medicine resistance and, in some instances, improve the therapeutic response rate. To effectively implement these tactics, one needs to have a thorough understanding of resistance mechanisms and the capability to manage therapy time and dosages appropriately. Therefore, additional research in this area is essential to develop strategies to successfully address the challenges of medicine resistance and improve patient therapy management [14, 162, 163]. During the initial stages of pharmacological treatment, there is a noticeable activation of concurrent signaling pathways, including YAP and PI3K, an elevation of anti-apoptotic proteins such as MCL1, and an enhancement of critical receptors like IGF1R, EGFR, MET, and AXL. Consequently, it is feasible to further reduce the number of remnant tumor cells by integrating targeted therapies that obstruct these pathways. Integrating targeted therapies with radiation, immunotherapy, or chemotherapy can enhance outcomes. Enhancing treatment approaches for individual patients and carefully adjusting pharmaceutical administration is essential for achieving optimal results and reducing unpleasant effects [164, 165].

#### Phenotypic modulation

A crucial strategy in combating drug resistance is to concentrate on significant crossings. Slow-cycling cells are recognized as a critical phase in the phenotypic transformation process, a fact that has been previously established. Research has shown that multiple routes facilitate the reinforcing of drug-resistant cells. These processes include enhanced IGF1R signaling, increased drug efflux, endoplasmic reticulum stress signaling, and intricate chromatin remodeling. The robust link between epigenetic aberrations and the incidence of DTPs suggests that a viable method may entail the development of pharmaceuticals that precisely target epigenetic regulators. Research has shown that H3K9me3 can facilitate changes, including the formation of heterochromatin in NSCLC [166]. This alteration results in the inhibition of long interspersed nuclear element 1 (LINE-1) and a reduction in the production of genes stimulated by antiviral response elements and Interferon. These epigenetic modifications augment the viability of DTPs. The reactivation of LINE-1 elements can be accomplished by inhibiting HDAC with agents such as trichostatin A or enthiostat (MS-275), reducing drug-resistant cell populations. Trichostatin A can efficiently eradicate drug-resistant cells by inhibiting LINE-1 elements. Studies have shown that trichostatin A treatment for melanoma cells characterized by slow-cycling and drug resistance yields a temporary positive effect. The survival of DTPs is greatly affected by the activity of histone demethylases KDM6A/B and KDM5A/B. In glioblastoma, stable cells exhibited reduced proliferation when KDM6 enzymatic activity was suppressed with a specific inhibitor. Comprehensive testing is essential to evaluate the properties and efficacy of the many KDM inhibitors developed. The transcriptional regulation of critical genes may be crucial for DTP survival via a transcriptional addiction mechanism, as these proteins are influenced by global chromatin changes that govern transcription. In T-cell acute lymphoblastic leukemia, cells produced after γ-secretase inhibitor therapy exhibit increased repressive histone marks and enhanced compaction of modified chromatin. The injection of JQ1 diminished cell viability by inhibiting the expression of essential genes, including BCL2 and MYC. Furthermore, interfering with the signaling pathways essential for establishing a new identity may impede MRD development and reduce the probability of resistance [167–170].

#### Restoring cellular identity

Epigenetic pathways often affect cellular plasticity; pharmacological therapies can mitigate this plasticity and reestablish cellular sensitivity. Nonetheless, inhibiting epigenetic regulators vital for cellular plasticity must be cautiously approached due to their considerable physiological importance. Thalidomide analogs can degrade SOX factors, critical transcription factors implicated in reprogramming. Given that these factors are closely linked to their chromatin environment, one alternative strategy is to suppress lineage-specific transcription factors by targeting chromatin-modifying enzymes associated with those factors. Moreover, multiple efforts have been made to inhibit TGF signaling to reverse the EMT [171]. Nonetheless, therapeutically targeting this pathway is challenging due to the myriad and complex roles TGFs fulfill in cancer, especially their dual participation at various stages of carcinogenesis. This complexity underscores the necessity for further research on the strategic use of TGF inhibitors in combination therapies, particularly to mitigate drug-induced cellular plasticity. Research has predominantly concentrated on inhibiting TGF-β signaling. Nonetheless, the intricate and paradoxical roles of TGF-β in cancer throughout different stages of tumor progression complicate the effective inhibition of this pathway for therapeutic applications. Therefore, it is essential to rigorously evaluate the effectiveness of TGF-β inhibition in combination therapies to reduce the impact of drug-induced cellular plasticity. Forskolin and cholera toxin have been found to activate the E-cadherin promoter in mesenchymal cells. These chemicals upregulate the MET pathway in mesenchymal cells, enhancing the cells’ vulnerability to anticancer agents [172].

#### Cellular reprogramming

Addressing cellular plasticity necessitates prioritizing innovative cell identities exhibiting drug resistance. This methodology may be executed with existing methodologies or identifying new vulnerabilities. Numerous studies are investigating the relationship between cancers exhibiting neuroendocrine differentiation and their treatment sensitivity profiles, which are analogous to minor cell variants [173]. SCLC cells originating from EGFR-mutant adenocarcinomas undergo a transition that leads to the cessation of EGFR expression, hence conferring resistance to EGFR inhibitors. Nonetheless, these cells exhibit a transient response to platinum-etoposide therapy, analogous to primary SCLC cells [174].

Studies suggest that specific patients with enzalutamide-resistant prostate cancer may exhibit remarkable responsiveness to pembrolizumab, a PD1 inhibitor. The link between AXL receptor tyrosine kinase expression and the emergence of the EMT characteristic in NSCLC suggests that AXL may serve as a new target for therapeutic intervention. The concurrent injection of erlotinib, an EGFR inhibitor, with SGI-7079, an AXL inhibitor, improves the sensitivity of NSCLC mesenchymal cells to erlotinib [175]. Nonetheless, inhibiting AXL does not augment susceptibility to TKIs; instead, it exhibits synergy with antimitotic agents. The observed variances may be ascribed to the lack of selectivity in existing AXL inhibitors. Research indicates that persons with NSCLC can safely administer BGB324, which preferentially targets AXL. In specific instances, it has even stabilized the condition. An increase in AXL expression has been observed in melanoma after pharmacological therapy. The simultaneous delivery of a therapeutic antibody and MAPK inhibitors to AXL-expressing cells has demonstrated efficacy in inhibiting tumor progression in xenograft models derived from melanoma patients [176, 177].

#### Autophagy and senescence in cancer cell plasticity

Autophagy and senescence are essential mechanisms that enhance cancer cell adaptability and confer treatment resistance. Autophagy is a survival mechanism during stressful conditions, such as exposure to chemotherapeutic drugs or targeted therapy. Autophagy facilitates metabolic adaptability by digesting damaged organelles and recycling intracellular components, enabling cancer cells to sustain survival in adverse conditions. Research indicates that autophagy suppression can enhance tumor sensitivity to treatment and interfere with phenotypic plasticity, especially in DTP cells. Senescence, viewed solely as a tumor-suppressive mechanism due to its capacity to induce sustained cell cycle arrest, is now acknowledged as a factor in therapy resistance and tumor growth through the senescence-associated secretory phenotype (SASP). SASP components, including IL-6, IL-8, MMPs, and growth factors, provide a pro-inflammatory and pro-tumorigenic milieu that fosters tumor heterogeneity, immune evasion, EMT, and the reprogramming of adjacent non-senescent cells into more plastic or stem-like states.

## Future directions

The plasticity of cancer cells and the shift from temporary drug tolerance to enduring resistance are fundamentally based on the interplay between epigenetic and metabolic reprogramming. Metabolites serve as crucial cofactors and direct regulators of epigenetic enzymes, while epigenetic modifications subsequently alter significant metabolic pathways, including glycolysis, fatty acid oxidation, and glutaminolysis. The bidirectional interplay guarantees that drug-tolerant persister cells cannot be eradicated by a singular intervention, as the epigenetic–metabolic network offers several redundant pathways for survival. Future perspectives should concentrate on strategies that concurrently address this common interface. Approaches may involve identifying and inhibiting “dual-function nodes” like EZH2 or BRD4, which control chromatin structure and metabolic activity; designing combination therapies that pair epigenetic inhibitors (e.g., HDAC or BET inhibitors) with metabolic inhibitors (e.g., LDHA or CPT1A blockers); and employing advanced delivery systems such as hypoxia-responsive nanocarriers or tumor-activated prodrugs to improve treatment specificity and efficacy. In addition to these strategies, several critical questions warrant further exploration: What epigenetic and metabolic signatures can be utilized as reliable biomarkers for the stratification of patients? Does sequential therapy, involving epigenetic priming followed by metabolic inhibition and standard treatment, provide greater advantages than concurrent administration? What is the role of exosomes and tumor-derived non-coding RNAs in restoring plasticity following dual inhibition? To address these questions, integrated multi-omics approaches, systems-level modeling, and patient-tailored clinical trials are necessary. These efforts will enhance our comprehension of the fundamental mechanisms underlying plasticity and resistance, while also expediting the advancement of more effective and durable therapeutic strategies. The growing conjunction of artificial intelligence (AI), machine learning (ML), and systems biology must be admitted as ground-breaking tools for understanding and predicting the course of cancer.These computational approaches promote the analysis of complicated, high-dimensional datasets acquired from genomes, transcriptomics, proteomics, and imaging, hence facilitating precise modeling of tumor dynamics and resistance patterns. AI systems and deep learning models have shown great promise in anticipating drug reactions, detecting resistance-related biomarkers, and classifying patients for improved treatment protocols.Simultaneously, systems biology enables the simulation of complex molecular networks, the reconstruction of signaling pathways, and the identification of susceptibility nodes within plasticity-related circuits, including EMT, CSC maintenance, and epigenetic control. Integrating AI and systems biology creates a framework for developing predictive digital twins. These individualized computational models of malignancies may simulate responses to diverse treatment regimens before clinical implementation. This convergence enhances precision oncology and presents a proactive strategy for therapy design, intending to address resistance preemptively before its clinical emergence.

## Conclusion

Cancer cell plasticity serves as an evolutionary adaptation to therapeutic selection pressures and microenvironmental stresses, offering a dynamic strategy that enhances survival probability. Tumors increase the likelihood of subclones that can adapt to therapy by generating a spectrum of phenotypic states. Plasticity facilitates evasion from drug pressure, hypoxia, nutrient deprivation, and host immune surveillance. The emergence of transient drug-tolerant states, which may evolve into stable drug-resistant phenotypes, is driven by the synergistic interplay of signaling networks, epigenetic remodeling, metabolic reprogramming, and intercellular communication. The tumor microenvironment comprises various stromal and immune cells, including cancer-associated fibroblasts, M2-polarized macrophages, regulatory T cells, endothelial cells, and mesenchymal stem cells, which secrete critical factors such as TGF-β, TNF-α, IL-6, and VEGF. These signals enhance EMT, stemness, and survival pathways. TGF-β induces EMT via both SMAD-dependent and independent pathways, while also collaborating with the Hippo/YAP–TAZ axis to enhance invasive transcriptional programs. IL-6 activates STAT3 signaling to maintain stemness and tolerance; TNF-α triggers NF-κB to enhance survival and inflammation; and VEGF stabilizes HIF-1α, promoting angiogenesis, metabolic adaptation, and EMT. Extracellular matrix stiffness and mechanotransduction through integrin FAK–SRC signaling increase nuclear YAP/TAZ activity, establishing a feed-forward loop that perpetuates EMT and invasive behavior. The interaction among key oncogenic pathways, such as Wnt/β-catenin, Notch, TGF-β, Hedgehog, PI3K/AKT/mTOR, and RAS–ERK, contributes to the stabilization of plastic states. The Wnt and Notch pathways collaborate to preserve stem-like characteristics, while TGF-β works in conjunction with YAP/TAZ to enhance EMT. Additionally, the PI3K/AKT pathway facilitates survival during therapy-induced stress and promotes protein translation and biomass accumulation in DTP cells via mTOR activation. Persister cells generally emerge from reversible, non-genetic alterations, including chromatin remodeling and metabolic switching. Under sustained therapeutic pressure, these cells may evolve toward genetically fixed resistance. The fundamental mechanism underlying plasticity is predominantly epigenetic. Changes in chromatin accessibility, histone modifications (such as H3K27 trimethylation by EZH2 or histone deacetylation by HDACs), DNA methylation by DNMTs, and the remodeling of chromatin complexes like SWI/SNF play a dynamic role in regulating the promoters and enhancers of EMT and stemness genes. This plasticity allows for swift transitions among epithelial, mesenchymal, and hybrid states without necessitating immediate mutations, thus promoting early drug tolerance. Metabolic reprogramming concurrently sustains these states: glycolytic switching, known as the Warburg effect, generates ATP and biosynthetic precursors; fatty acid oxidation ensures energy stability during stress; glutamine metabolism supports the TCA cycle; and redox balancing mitigates harmful ROS while preserving NAD+/NADH ratios, thereby improving tolerance to therapies that induce oxidative stress. Intercellular communication introduces an additional regulatory layer. Exosomes and extracellular vesicles containing miRNAs, lncRNAs, proteins, and lipids transmit plasticity signals throughout tumor tissues. These vesicles facilitate the transfer of EMT and stemness programs from resistant to sensitive cells, activate survival pathways such as PI3K/AKT and Wnt in recipient cells, and deliver immune checkpoint molecules like PD-L1 to enhance local immune suppression. These exchanges contribute to the homogenization of resistant phenotypes and elevate the risk of disease relapse following an initial response.

## Data Availability

No datasets were generated or analysed during the current study.
